# Differences of skin carotenoids and adherence to the Mediterranean Diet pattern in adults from Southern Italy and Dominican Republic

**DOI:** 10.1186/s12967-024-05224-5

**Published:** 2024-05-04

**Authors:** Giuseppina Augimeri, Manuel Soto, Fabrizio Ceraudo, Giovanna Caparello, Melisa Villegas Figueroa, Mirko Cesario, Lorenzo S. Caputi, Berniza Calderón, Daniela Bonofiglio

**Affiliations:** 1 Department of Pharmacy, Health and Nutritional Sciences, University of Calabria, 87036 Arcavacata di Rende (CS), Italy; 2 Research Unit, Centro Médico de Diabetes, Obesidad y Especialidades (CEMDOE), Clara María Pardo Street, Santo Domingo, 10135 Distrito Nacional, Dominican Republic; 3 Instituto Tecnológico de Santo Domingo (INTEC), Los Proceres Avenue, Santo Domingo, 10602 Distrito Nacional, Dominican Republic; 4 UNICARIBE Research Center, University of Calabria, 87036 Rende, Italy; 5 Surface Nanoscience Group, Department of Physics, University of Calabria, 87036 Rende, Italy; 6 Sociedad Dominicana de Endocrinología y Nutrición (SODENN), 157 Independencia Avenue, GS Professional Building, Santo Domingo, 10206 Distrito Nacional, Dominican Republic; 7 Centro Sanitario, University of Calabria, Via P. Bucci, Arcavacata Di Rende (CS), 87036 Rende, Cosenza Italy

**Keywords:** Veggie Meter®, Mediterranean Diet, Carotenoid score, Fruit and vegetable intake

## Abstract

**Background:**

The measurement of the skin carotenoids using the Veggie Meter® has emerged as a rapid objective method for assessing fruit and vegetable intake, highly recommended by the Mediterranean Diet (MD), which represents one of the healthiest dietary patterns, worldwide. This study aimed to examine differences in skin carotenoid content and degree of adherence to the MD pattern between two adult populations from Southern Italy and the Dominican Republic.

**Methods:**

This cross-sectional study enrolled a total of 995 adults, 601 subjects from Italy and 394 from the Dominican Republic. All participants underwent anthropometric measurements and skin carotenoid assessment by Veggie Meter®. Adherence to the MD and lifestyle were evaluated using the Mediterranean Diet Adherence Screener (MEDAS) and the Mediterranean Lifestyle Index (MEDLIFE) questionnaires. Correlations between the skin carotenoid and MEDAS score were estimated using Pearson’s correlation coefficient. Multiple linear regression models were created to determine variables that affect skin carotenoid score for both populations.

**Results:**

Mean total skin carotenoids were higher in the Italian compared to the Dominican Republic population (342.4 ± 92.4 *vs* 282.9 ± 90.3; p < 0.005) regardless of sex (women: 318.5 ± 88.9 *vs* 277.3 ± 91.9, p < 0.005 and men: 371.7 ± 88.3 *vs* 289.5 ± 88.1, p < 0.005), and remaining statistically significant after age-adjustment of the Dominican Republic sample. Using the MEDAS questionnaire, we found a higher MD adherence score in the Italian than in the Dominican Republic population also after age-adjusting data (7.8 ± 2.1 *vs* 6.2 ± 3.7; p < 0.005) and even when categorized by sex (Italian *vs* age-adjusted Dominican Republic women: 7.9 ± 2.1 *vs* 6.3 ± 2.6; Italian vs age-adjusted Dominican Republic men: 7.7 ± 2.2 *vs* 6.0 ± 4.7; p < 0.005). Using the MEDLIFE test, total Italians presented a lower score with respect to the age-adjusted Dominican Republic population (3.2 ± 1.2 *vs* 3.4 ± 1.4; p < 0.05). In multiple regression analysis, skin carotenoids were associated with sex and negatively associated with BMI in the Italian population (sex: β: 54.95; 95% CI: 40.11, 69.78; p < 0.0001; BMI: β: − 1.60; 95% CI: − 2.98,0.86; p = 0.03), while they resulted associated with age and sex in the Dominican Republic population (age: β: 2.76; 95% CI: 1.92, 3.56; p < 0.001; sex: β: 23.29; 95% CI: 5.93, 40.64; p = 0.009). Interestingly, skin carotenoids were positively correlated with MEDAS score in both populations (Italy: r = 0.03, p < 0.0001, Dominican Republic: r = 0.16, p = 0.002).

**Conclusions:**

This study provides the assessment of the adherence to the MD and skin carotenoid content in adults living in Southern Italy and the Dominican Republic, showing a higher MD adherence score and a skin carotenoid content in inhabitants from the Mediterranean region. Our findings highlight the need to globally encourage fruit and vegetable intake, particularly in non-Mediterranean area.

## Introduction

Health and well-being are influenced by several factors, including lifestyle choices such as the consumption of a healthy diet and regular physical activity. These lifestyle choices are essential for the prevention and treatment of metabolic and chronic diseases throughout a person’s lifetime [1, 2]. Based on these concepts, many studies have reported that the Mediterranean Diet (MD) pattern is one of the most effective models promoting overall health [3, 4].

The MD pattern originated in the civilizations surrounding the Mediterranean Sea, consists of a high intake of plant-based foods, especially fruits and vegetables, olive oil, legumes, cereals, nuts, minimally processed local foods, moderate consumption of wine, poultry, fish and dairy products, and low consumption of red meat and sweets [4, 5]. Other Mediterranean lifestyle recommendations include physical activity and spending leisure time with friends and family [4]. Reduced risks for cardiovascular disease, overweight and obesity, diabetes, metabolic syndrome, cancer, cognitive decline, and total mortality are recognized as the main health benefits [4, 6, 7]. Interestingly, the beneficial effects of the MD are, at least in part, associated with the fruit and vegetable intake [8], highlighting the importance of ensuring an adequate intake of these foods to reduce the risk of chronic diseases in the population.

Even though it has been proven as one of the healthier dietary patterns, adherence to the MD varies greatly according to geographical location, even within the same country. Food choice availability, climate, affordability, personal nutritional preferences, and awareness are all factors that influence adherence to the MD, especially in non-Mediterranean countries [9, 10]. Furthermore, recent rapid changes in societies around the world due to globalization and urbanization have affected lifestyle choices, causing an increase in the consumption of processed foods and sedentarism [11–13]. Consequently, a reduction in the adherence to the MD and a shift toward a Westernized diet characterized by high intakes of fat and sodium, occurred even in the Mediterranean countries over the last decades. In this context, the global promotion of a healthy and sustainable dietary model based on nutritional properties and key concepts of MD represents a challenge set by the United Nations in the Agenda 2030 to decrease the burden of non-communicable diseases and their risk factors [9]. Among the tools to assess the adherence to the MD, the administration of food or lifestyle questionnaires, including the Mediterranean Diet Adherence Screener (MEDAS) and Mediterranean Lifestyle Index (MEDLIFE) questionnaires [14, 15], is one of the most used validated method to establish the degree of the adherence to the MD pattern. Recently, it has also been reported that the skin carotenoid content measured by the Veggie Meter®, a pressure-mediated reflection spectroscopy-based device which allows an easy and rapid estimation of the fruit and vegetable intake, is associated with the MD adherence score, representing an objective method to assess the compliance to the MD avoiding bias due to the utilization of self-reported questionnaires [16]. This study, in the context of the UNICARIBE project, aims to measure the skin carotenoid score by Veggie Meter® and to assess the adherence to the MD pattern, using MEDAS and the MEDLIFE questionnaires, in two populations living in a Mediterranean and non-Mediterranean country represented by Southern Italy and Dominican Republic.

## Material and methods

### Study design

In this cross-sectional study, a total of 995 adults were enrolled, 601 subjects from the Italian and 394 from the Dominican population, as part of UNICARIBE project which aims to develop joint research programs between the University of Calabria Italy and Caribbean research institutions. Data were collected from September 2023 to October 2023. In Italy, subjects were recruited at the University of Calabria, while Dominican subjects were recruited at the Instituto Tecnológico de Santo Domingo (INTEC) and at the Centro Médico de Diabetes, Obesidad y Especialidades (CEMDOE). Considered inclusion criteria were being older than 18 years of age living in Southern Italy or having Dominican Republic nationality. Subjects who had liver diseases were excluded from the study. Written informed consent was obtained from all participants, and they were then asked to complete questionnaires to assess their adherence to the MD and lifestyle. After completing the questionnaires, participants were subjected to anthropometric and skin carotenoid measurements.

### Assessment of adherence to MD and lifestyle

The adherence to the MD and lifestyle was assessed with the Mediterranean Diet Adherence Screener (MEDAS) (Table 6) and the Mediterranean Lifestyle Index (MEDLIFE) (Table 7) questionnaires respectively. The Italian and Spanish versions of each of these questionnaires were used for each respective population.

The MEDAS is a validated questionnaire that comprises 14 items that evaluate the intake of main group foods consumed in the MD. Responses that are inside the MD recommendations are given a score of 1, while responses outside the recommendations are given a score of 0. Scores for each item are added together and a total score that ranges from 0 to 14 is obtained. Total scores ranged 0–5 are considered low adherence, scores ranged 6–9 are medium adherence and scores ≥ 10 are good adherence to the MD [14, 17].

The MEDLIFE is a validated questionnaire that comprises 28 items, divided into 3 blocks, that evaluates the adherence to both Mediterranean food consumption and lifestyle habits. Block 3 consists of 6 items that specifically evaluate lifestyle habits, this was the part of the questionnaire that was used to assess the adherence of participants to the Mediterranean lifestyle. Responses that are consistent with the Mediterranean lifestyle are given a score of 1, while others are given a score of 0. Items’ scores are added together for a range of total score from 0 to 6, with higher scores representing a higher adherence to the Mediterranean lifestyle [15].

### Anthropometric measurements

Weight, height, and waist circumference were measured by trained personnel using standardized procedures. Weight and height were measured using a previously calibrated analogue scale. Waist circumference was measured using an anthropometric measuring tape. Body Mass Index (BMI) was calculated using the standard BMI formula [18].

### Skin carotenoid measurement

Levels of carotenoid in the skin were measured using the Veggie Meter®, which uses light spectroscopy for measurements. Measurements were performed according to the manufacturer’s instructions. Before performing any measurements, the device was properly calibrated. Skin carotenoid level measurements were performed by applying light pressure on the participant’s index finger. The device’s scores range from 0 to 800, with higher scores indicating higher skin carotenoid concentrations [19, 20].

### Sample size and power calculation

Sample size calculation was performed using a 95% confidence interval (CI) and a margin of error (d) of 5% for both the Italian and Dominican populations. An expected adherence to the Mediterranean diet (P) of 50% was used to obtain the maximum possible sample size using the following formula:

$$n = \frac{{Z^{2} P(1 - P)}}{{d^{2} }}$$

A minimum sample size of 385 participants per country was obtained. Due to a high participation rate, a total of 601 participants were recruited in Italy and 394 were recruited in the Dominican Republic. Post-hoc power calculations for statistical differences between means of the two populations (with two-tailed Student’s t-test) for small effect size (Cohen’s D = 0.2) yielded a statistical power (β) of 86%.

### Statistical analysis

Quantitative variables were reported as mean and standard deviation (SD), while categorical variables were reported with absolute frequency and percentage. The Dominican Republic sample results were sex and age-adjusted to the Italian sample by calculating weighted averages. Weights for the Dominican population were calculated by dividing the proportion of individuals for each age group for both sexes of the Italian sample by the proportions in the Dominican sample. The age groups used for the analysis were: from 18 to 30 years, 31 to 40 years, 41 to 50 years, 51 to 60 years, and older than 60 years.

Statistically significant differences between the Italian and Dominican Republic populations were evaluated using the Student’s T-test with Welch’s correction, where applicable. Correlations between the skin carotenoid and MEDAS score were evaluated using Pearson’s correlation coefficient. Multiple linear regression models using the Ordinal Least Squares (OLS) method were created for both the Italian and Dominican populations to determine variables that affect skin carotenoid score and the variance inflation factor (VIF) was evaluated to assess multicollinearity among the predictor variables. Statistical analyses were performed using JASP and GraphPad-Prism 9 software programs. Statistical significance was established as a *p*-value < 0.05.

## Results

### General characteristics and carotenoid score of the population sample

Table 1 shows the general characteristics and the carotenoid score of our population sample. A total of 995 subjects from Southern Italy and the Dominican Republic were enrolled in this study. The mean age was significantly higher in the total Italian respect to the Dominican Republic population (27.4 ± 11.7 *vs* 25.7 ± 11.23, p < 0.05) and in men (28.4 ± 12.7 *vs* 23.5 ± 9.4, p < 0.001), but not in women. The mean BMI was higher in the total Dominican Republic than in the Italian population (25.1 ± 4.7 *vs* 22.7 ± 3.8, p < 0.001), regardless of the sex (women: 25.2 ± 5.2 *vs* 21.8 ± 3.3, p < 0.005, and men: 24.9 ± 4.1 *vs* 23.9 ± 3.9, p < 0.05). Similarly, waist circumference was significantly higher in the total Dominican Republic than in the Italian population sample (81.2 ± 11.7 *vs* 78.3 ± 11.2, p < 0.005). Categorizing the population by sex, Dominican Republic women showed significantly higher waist circumference than Italian women (79.2 ± 12.1 *vs* 73.1 ± 8.6, p < 0.005), whereas no differences were found between the male population. Anthropometric parameters (BMI and waist circumference) were significant higher in the men than woman Italian (BMI: 23.9 ± 3.9 *vs* 21.8 ± 3.3, p < 0.0001; waist circumference: 84.8 ± 10.2 *vs* 73.1 ± 8.6, p < 0.0001), whereas no gender-related differences were found in the Dominican Republic population. Interestingly, the carotenoid score in the total Italian population was significantly higher than in the Dominican Republic sample (342.4 ± 92.4, *vs* 282.9 ± 90.3, p < 0.005) regardless of the sex (women: 318.5 ± 88.9 *vs* 277.3 ± 91.9, p < 0.005 and men 371.7 ± 88.3, *vs* 289.5 ± 88.1, p < 0.005). In addition, we found a higher carotenoid content in the Italian men than in women (371.7 ± 88.3 *vs* 318.5 ± 88.9, p < 0.0001), while no gender-related differences were found in the Dominican Republic population.

**Table 1:** General characteristics and carotenoid score of the Italian and the Dominican Republic population

Characteristics	Italy	Dominican Republic (unadjusted)	Dominican Republic (age-adjusted)
Subjects (n)	601	394	
Women (n)	331	213	
Men (n)	270	181	
Age (yrs)	27.4 ± 11.7	25.7 ± 11.23 *	26.2 ± 24.6
Women	26.6 ± 10.7	27.7 ± 14.6 ^#^	25.8 ± 7.6
Men	28.4 ± 12.7	23.5 ± 9.4 ***	26.6 ± 35.4
BMI (Kg/m^2^)	22.7 ± 3.8	25.1 ± 4.7 ***	25 ± 10.7 ***
Women	21.8 ± 3.3^§^	25.2 ± 5.2 ***	24.8 ± 5.7 ***
Men	23.9 ± 3.9	24.9 ± 4.1 *	25.2 ± 14.6
Waist circumference (cm)	78.3 ± 11.2	81.2 ± 11.7 ***	81.3 ± 38.4
Women	73.1 ± 8.6^§^	79.2 ± 12.1 ***, ^•^	78.1 ± 14.5 ***
Men	84.8 ± 10.2	83.6 ± 10.8	85.2 ± 54.2
Carotenoid Score	342.4 ± 92.4	282.9 ± 90.3 ***	283.0 ± 158.4 ***
Women	318.5 ± 88.9^§^	277.3 ± 91.9 ***	273.0 ± 95.1 ***^,#^
Men	371.7 ± 88.3	289.5 ± 88.1 ***	294.9 ± 209.5 ***

Values are mean ± standard deviation. *p*-values were obtained using Student T-test. *p < 0.05; ***p < 0.0005; §: women vs men *p* value: < 0.0001; # women *vs* men, *p*:0.001; •waist circumference for women *vs* men *p*:0.0002; n: number; yrs: years; BMI: Body Mass Index

Importantly, BMI, waist circumference and carotenoid score remained statistically significant after the age-adjustment of the Dominican Republic population sample.

### Associations between carotenoid score and different variables in both Italian and Dominican Republic participants

Multiple regression analysis was performed using carotenoid score as independent variable and age, sex and BMI as dependent variables in both Italian and Dominican Republic populations. The results showed that carotenoid score was positively associated with sex in the Italian population (p < 0.0001) and negatively associated with BMI (p = 0.03), while a significant positive relationship with sex (p = 0.009) and age (p < 0.001) was found in the Dominican Republic sample (Table 2). Our regression analysis revealed low explained variances for Italy and the Dominican Republic (Italy: Adj. R^2^ = 0.080, Dominical Republic: Adj. R^2^ = 0.098), while the VIF didn’t evidence multicollinearity among age, BMI and sex (data not shown).

**Table 2:** Multiple regression analysis among Carotenoid score and different variables in the Italian and Dominican Republic population sample

	Italy		Dominican Republic	
	β (95% CI)	p-values	β (95% CI)	p-values
Age	0.26 (− 0.36, 0.88)	0.41	2.74 (1.92, 3.56)	< 0.001
BMI	-1.60 (-2.98,0.86)	0.03	− 1.74 (− 3.68, 0.19)	0.077
Sex (M)	54.95 (40.11, 69,78)	< 0.0001	23.29 (5.93, 40.64)	0.009
Adj. R^2^	0.080		0.098	

*BMI* Body Mass Index, *Adj* Adjusted, *CI* Confidence Interval

### Adherence to the Mediterranean diet in both Italian and Dominican Republic participants

The MD recommends high intakes of fruits and vegetables, which are enriched with carotenoids. To date, adherence to the MD pattern is assessed through the administration of diet questionnaires, including the Mediterranean Diet Adherence Screener (MEDAS). In addition, the Mediterranean Lifestyle Index (MEDLIFE) questionnaire allows a rapid and comprehensive estimation of adherence to the MD lifestyle [14, 21]. Using the MEDAS test, we observed an average degree of adherence to the MD in both populations. However, the Italian population showed significantly higher MD scores compared to the unadjusted or age-adjusted Dominican Republic population (7.8 ± 2.1 *vs* 6.1 ± 2.3 and 7.8 ± 2.1 *vs* 6.2 ± 3.7, p < 0.001, respectively), even when categorized by sex (women: 7.9 ± 2.1 *vs* 6.4 ± 2.3 and 7.9 ± 2.1 *vs* 6.3 ± 2.6, p < 0.001, respectively; men: 7.7 ± 2.2 *vs* 5.8 ± 2.3 and 7.7 ± 2.2 *vs* 6.0 ± 4.7, p < 0.001, respectively). In contrast, using the MEDLIFE test, adherence to the MD pattern was lower in the Italian than in the unadjusted or age-adjusted Dominican Republic population (3.2 ± 1.2 *vs* 3.4 ± 1.2 and 3.2 ± 1.2 *vs* 3.4 ± 1.8, p < 0.05, respectively), whereas no differences were observed when categorizing population by sex after adjusting for age (Table 3). In contrast, we observed higher MEDLIFE score in the men than women Italian population (3.5 ± 1.2 *vs* 3.0 ± 1.2), while significant differences were found between sexes for the MEDAS score and MEDLIFE score in the Dominican population (women *vs* men: 6.4 ± 2.3 *vs* 5.8 ± 2.3, p = 0.01; women *vs* men: 3.1 ± 1.1 *vs* 3.8 ± 1.2, p = 0.01, respectively).

**Table 3:** Mediterranean Diet Adherence Screener (MEDAS) and Mediterranean Lifestyle index (MEDLIFE) score in the total population sample or categorized by sex

	Italy	Dominican Republic (unadjusted)	Dominican Republic (age-adjusted)
MEDAS score			
Total	7.8 ± 2.1	6.1 ± 2.3***	6.2 ± 3.7***
Women	7.9 ± 2.1	6.4 ± 2.3***	6.3 ± 2.6***
Men	7.7 ± 2.2	5.8 ± 2.3***	6.0 ± 4.7***
MEDLIFE score			
Total	3.2 ± 1.2	3.4 ± 1.2*	3.4 ± 1.8*
Women	3.0 ± 1.2^§^	3.1 ± 1.1	3.1 ± 1.4
Men	3.5 ± 1.2	3.8 ± 1.2 *	3.7 ± 2.1

Values are mean ± standard deviation. *p*-values were obtained using Student T-test. *p < 0.05; ***p < 0.0005, ^§^Women vs Men p=0.0001

**Fig. 1:**
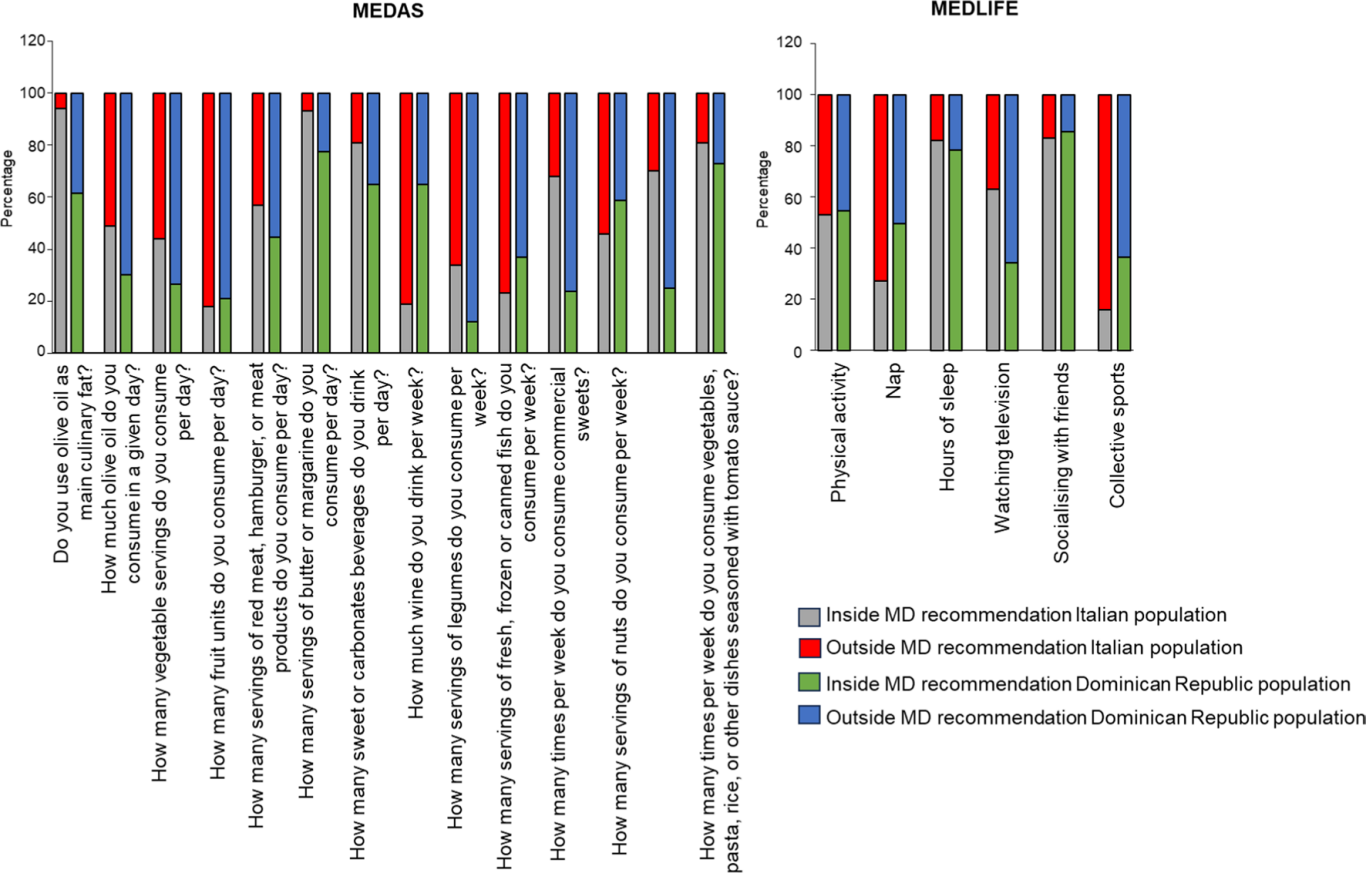
Compliance with items from Mediterranean Diet Adherence Screener (MEDAS) and the Mediterranean Lifestyle Index (MEDLIFE) questionnaires in the total sample of each population. Percentage distribution of each population with respect to the cut-off points within or outside recommendations according to MEDAS and MEDLIFE score. MD: Mediterranean Diet

The compliance rates for each MD recommendation using the MEDAS and MEDLIFE questionnaires in the total populations were calculated and shown in Fig. 1.

### Impact of the Mediterranean Diet food choices on the carotenoid score in Italian and Dominican Republic population

To evaluate the impact of the Mediterranean Diet food choices on the skin carotenoid score in the two populations, we selected specific items with highest carotenoid content from the MEDAS questionnaire, as follows: item 1: “*How many vegetable servings do you consume per day?*”; item 2: “*How many fruit units do you consume per day*?”; item 3:”*How many times per week do you eat dishes with a sauce of tomato, garlic, onion/leeks sautéed in olive oil?*”. Intriguingly, the carotenoid score of each item was significantly higher in the Italian than in the Dominican Republic sample (Table 4).

**Table 4:** Carotenoid score in the population categorized in different items from MEDAS

	Italy	Dominican Republic	*p-*values
ITEM 1			
Subjects (n)	264	105	
Carotenoid score	356.08 ± 94.52	293.36 ± 97.35	< 0.0001
ITEM 2			
Subjects (n)	106	83	
Carotenoid score	378.8 ± 99.62	278.17 ± 98.79	< 0.0001
ITEM 3			
Subjects (n)	487	217	
Carotenoid score	344.87 ± 93.10	285.83 ± 89.05	< 0.0001

Values are mean ± standard deviation. n: number. *p*-values between the Italian and Dominican Republic population were obtained using Student T-test with Welch’s correction

### Correlation between the Mediterranean Diet score and the skin carotenoid score

**Fig. 2:**
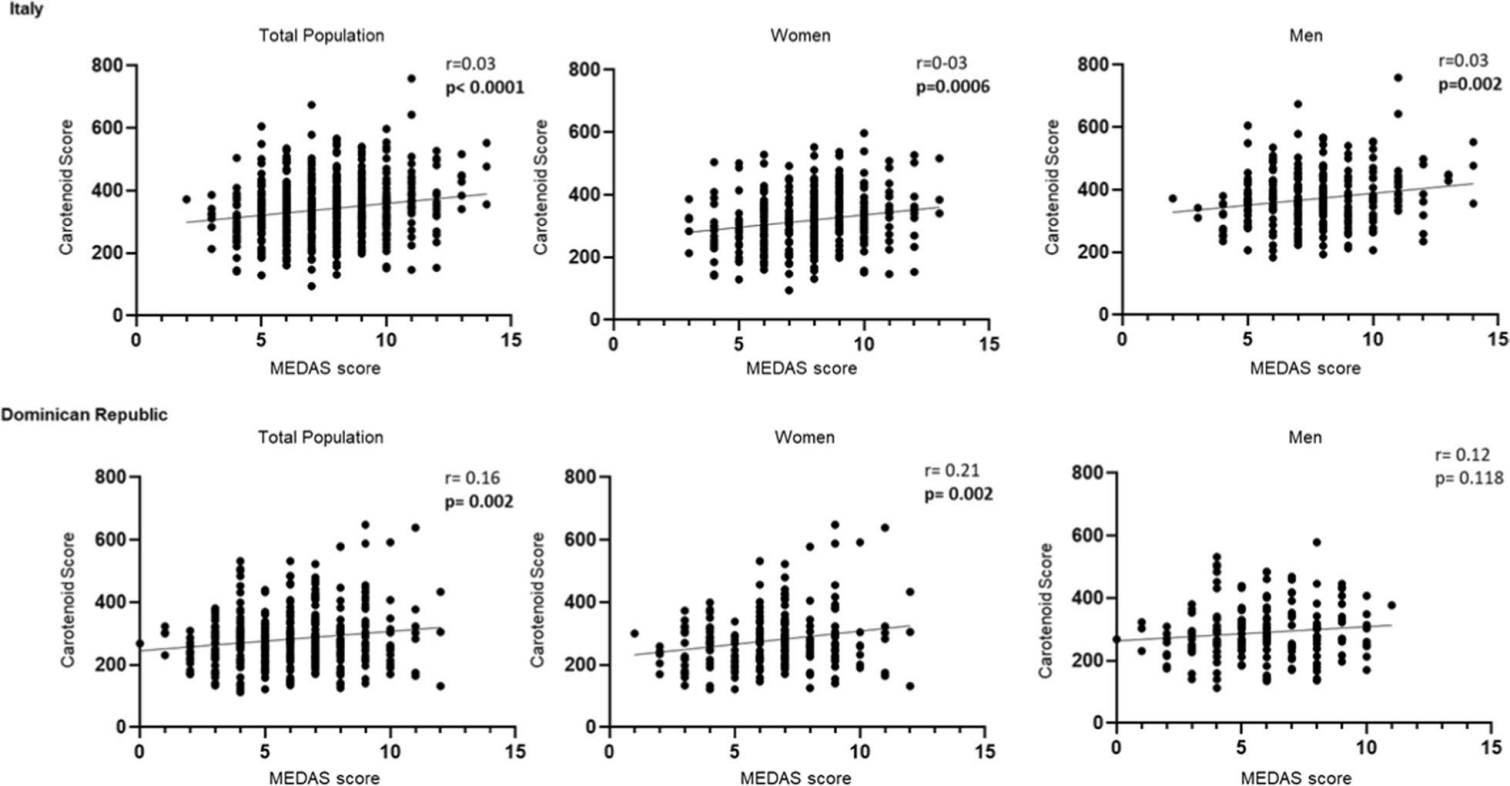
Correlations between carotenoid score and MEDAS score in the total population, in women and in men from Italy (upper panel) and Dominican Republic (lower panel). The association between skin carotenoid content and MEDAS score was analyzed using Pearson’s correlation test. For each linear regression graph, the correlation coefficient—r and the statistical significance—*p* are reported

The MEDAS questionnaire represents a validated tool to evaluate adherence to the MD. However, bias due to the self-reported intakes can occur impacting the accuracy of the results. To test whether the skin carotenoid score might be used as a potential parameter to evaluate the MD adherence in adults from different countries, we estimated the correlations between the MEDAS score and the carotenoid score by Pearson’s correlation in both the Italian and Dominican Republic populations. We observed that skin carotenoids are positively associated with MEDAS score in the total Italian population (p < 0.0001), in women (p = 0.0006) and men (p = 0.002) (Fig. 2, upper panel). Similarly, we found a positive association between skin carotenoids and MEDAS score in the total Dominican population (p = 0.002) and in women (p = 0.002) but not in men (Fig. 2, lower panel).

Finally, multiple linear regression analysis was performed using the carotenoid score as the dependent variable and the MEDAS score along with age, sex, BMI as independent variables, in both populations. We found that in both the Italian and Dominican Republic samples, carotenoid score was significantly associated with MEDAS score (p < 0.0001 and p = 0.03, respectively) and sex (p < 0.0001 and p = 0.005, respectively), while it was also correlated with age (p < 0.001) in the Dominican Republic population (Table 5). When other predictors were adjusted for, the MEDAS score led to significant increases in skin carotenoids levels of 8.34 in Italians and 4.23 in Dominicans, respectively. although our regression model yields with least explained variance (Italy: Adj. R^2^ = 0.112, Dominican Republic: Adj. R^2^ = 0.107).

**Table 5:** Multiple regression analysis among Carotenoid score, MEDAS and different variables in Italian and Dominican Republic population

	Italy		Dominican Republic	
	β (95% CI)	p-value	β (95% CI)	p-value
MEDAS score	8.34 (4.91, 11,76)	< 0.0001	4.23 (0.43–8.04)	0.03
Age	− 0.084 (− 0.71, 0541)	0.79	2.55 (1.72, 3.39)	< 0.001
BMI	− 1.43 (− 3.33, 0.47)	0.14	− 1.76 (− 3.69, 0.16)	0.07
Sex (M)	57.47 (42.86, 72.07)	< 0.0001	24.91 (7.57, 42.24)	0.005
Adj. R^2^	0.112		0.107	

## Discussion

The primary objective of our study was to measure the skin carotenoid content by Veggie Meter® and to compare the degree of adherence to the MD pattern in an adult population enrolled from two countries of the Mediterranean and non-Mediterranean area, represented by Italy and Dominican Republic, respectively.

Our results showed higher levels of skin carotenoids in the total Italian compared to the Dominican Republic population sample as well as in women and men, which remained statistically significant after the age-adjustment of the Dominican Republic population. Moreover, we observed a significantly higher carotenoid score in men than women in both Italian and Dominican population. The differences between the Italian and Dominican population suggest that carotenoids intake could be improved in the inhabitants of the Caribbean, as also reported in a previous study investigating the carotenoid levels in adults from the same geographic area [22]. Additionally, the National Demographic and Health Survey (ENDESA) reported that most adults have a low intake of fruit and vegetables, with only 85% reporting eating them regularly at least 1 to 2 times a week on average [23].

As expected, we also found that BMI and waist circumference were lower in total Italian sample than in inhabitants of Dominican Republic. Particularly, gender-related differences were found in Italian, but not in the Dominican Republic population. Our findings are in agreement with data of age-standardized estimate of mean BMI, overweight and obesity in Italy and Dominican Republic adults of both sexes in 2017 [24, 25]. A study conducted by Casperson et al. demonstrated that non-obese adults have a good level of skin carotenoids if their dietary consumption of fruits and vegetables intake is adequate [26]. Similarly, Caparello et al. have recently published that the skin carotenoid content was within the normal range and inversely related to the BMI in a cohort of healthy adolescents from a Mediterranean area [16]. However, conflicting data are reported on the correlation between BMI and carotenoid levels in the population depending on different factors including sex, age or age groups but also on total or individual carotenoids [27–29]. Particularly, it has been reported that BMI was inversely related to serum concentrations of total carotenoids in women, regardless of age categories. This correlation remained significant for individual carotenoids, such as lutein, zeaxanthin, β-cryptoxanthin, α-carotene, β-carotene, whereas it was statistically not significant for lycopene, suggesting that not all carotenoids exhibited the same properties [28]. Conversely, total blood carotenoid concentrations were negatively associated with BMI only in men aged 40–59 years, while individual carotenoids did not display any significant correlation with this anthropometric measurement [28]. Suzuki et al. also reported lower serum carotenoid levels and higher waist circumference values in adults, indicating an inverse relationship between visceral obesity and serum carotenoid content in a healthy Japanese population [29]. In a cross-sectional study carried out in a middle-aged healthy population, higher skin carotenoid content was associated with lower BMI in women and in men [27]. In addition, skin carotenoids were positively correlated with both total and individual serum carotenoid concentrations [27] confirming that skin carotenoids represent a valuable biomarker for the estimation of fruit and vegetable intakes.

In multiple regression analysis, we found an inverse correlation between carotenoids and BMI in the Italian, but not in the Dominican Republic populations highlighting the influence of this parameter on the carotenoid levels. Notably, we showed that skin carotenoid content directly correlated with men in both populations and with age in the Dominican Republic sample. These findings are suggestive that men were more likely to be well-compliant and that older participants who perceived themselves to be overweight or obese were more likely to adopt healthier eating habits. Indeed, a large body of evidence has reported that carotenoid levels are inversely associated with markers of metabolic and cardiovascular disease in healthy individuals, suggesting that fruit and vegetable intake may exert preventive effects against several chronic pathological conditions [27, 28, 30]. The MD, enriched with fruit and vegetables, is considered to be a model of gold-standard eating habits for its contribution to a healthy status and a better quality of life, and has been encouraged as part of healthy nutrition programs [31–33]. It has been largely reported that the geographical location and the economic, cultural and social features are crucial factors that may affect MD adherence [10]. Although the MD is traditionally followed by the populations of the Mediterranean basin, over the last decades, Mediterranean populations have been showing moderate adherence to the Mediterranean diet [17, 34–37], suggesting the need for improving MD adherence even in the countries of its origin.

Several food questionnaires are available to assess adherence to the MD, including the Medi-Lite developed in Italy [38]. In this study, we administered to our sample population the MEDAS questionnaire since it has been widely used and validated in various populations and has demonstrated good reliability in evaluating adherence to the MD [14, 17]. We found an average adherence to the food recommendations of the MD in both population samples, which was significantly higher in Southern Italy compared to the Dominican Republic. Regarding lifestyle habits, our research highlighted that the Italians presented a lower score with respect to the Dominican Republic population. Our results are in line with the survey that examined the lifestyle and the levels of physical activity in the Italian population, underlining the importance of suggesting an improvement in the active lifestyle, especially in Southern Italy [39–41].

Another important contribution of our study is that the carotenoid score was directly associated with adherence to the MD pattern in both populations, supporting the same results recently published in healthy adolescents from Southern Italy [16]. To evaluate the impact of MD food choices on the skin carotenoid content between the two population sample, we selected items from the MEDAS questionnaire accounting for fruits, vegetables and dishes with tomato sauce which are enriched in carotenoids. Interestingly, we observed that for each item carotenoid content was significantly higher in the Italian than Dominican Republic population. Therefore, we can hypothesize that even though the food choices mostly fall on plant-based foods they differently influenced the qualitative composition of carotenoids in local products of our populations from Mediterranean and non-Mediterranean countries.

A primary strength of this study is that we used an objective method to assess the skin carotenoid content in population samples living in different geographical areas. This method is well suited for adolescents and adults and makes it useful to perform wide scale screening and promotion campaigns for increasing fruit and vegetable intakes. Furthermore, this tool makes it possible to investigate food quality related to specific eating habits at the individual level. Moreover, our study provides a comparison of the general characteristics and MD behavioural nutrition and physical activity between the Italian and the Dominican Republic population sample, showing many differences among variables tested that deserve to be further investigated. Despite these strengths, there are some limitations to consider. First, all participants were volunteers and enrolled without specific exclusion criteria regarding health problems, any type of restrictive diet or digestive problems, or the use of drugs that could interfere with the absorption of carotenoids because this was not part of the ethical approval. Second, it's crucial to acknowledge that this study did not incorporate actual consumption data of participants, as adherence to the MD was evaluated by self-reported MD questionnaires. Although this method of data collection is a validated tool, it is inherently susceptible to measurement error. Moreover, some fruits or vegetables do not contain carotenoids and, therefore, the Veggie Meter® was unable to detect them. Lastly, most of the participants were enrolled from the university communities of both countries and some older Dominican subjects were recruited at a Health Center during a control visit, therefore generalizability of these findings may be limited, and data should be validated in a larger sample representative of all populations.

## Conclusions

In this study, we compared, for the first time to our knowledge, the measurements of skin carotenoids by Veggie Meter® and the adherence to the MD pattern in an adult population sample living in Italy and the Dominic Republic and we found healthier dietary habits in the Mediterranean than in non-Mediterranean area. In line with the goals of the Agenda 2030 and the ‘Planeterranean’ diet, our findings offer an objective basis for assessing the diet quality and for investigating the nutritional component in the plant foods typically recommended in MD for their health benefits and provide valuable input for national nutrition policies of both Mediterranean and non-Mediterranean countries.

## Figures and Tables

**Table 6:**
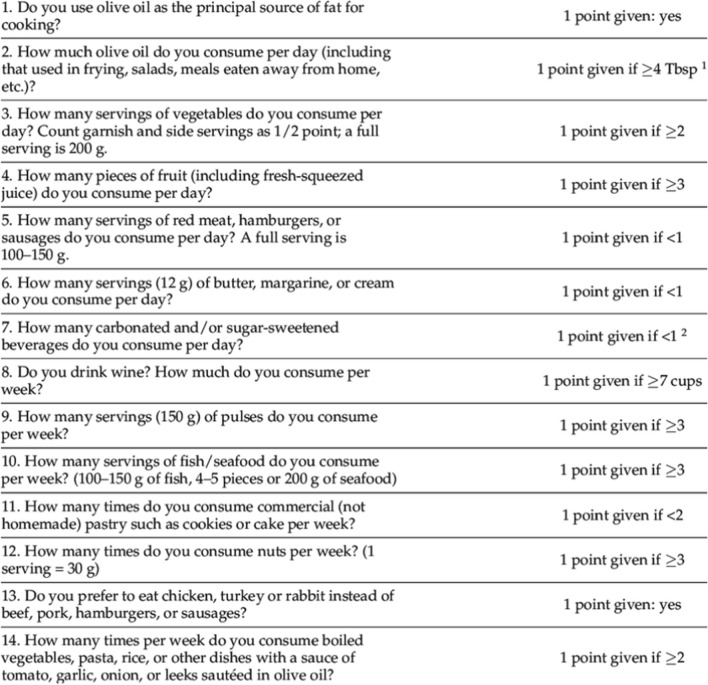
Mediterranean Diet Adherence Screener (MEDAS) questionnaire

**Table 7:**
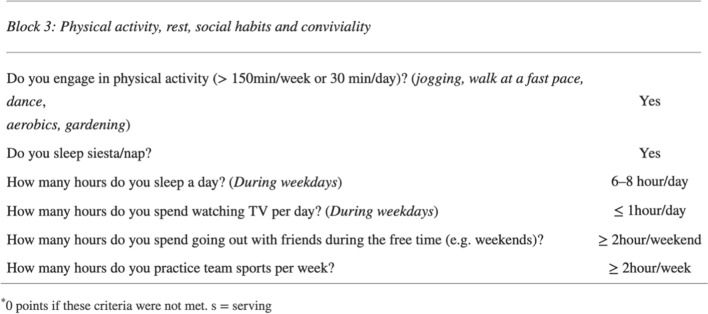
Mediterranean Lifestyle index (MEDLIFE) questionnaire. Block 3

## Data Availability

Please contact the corresponding author for data requests.

## Annexes

See Tables 6 and 7

## Abbreviations

BMI: Body mass index
CI: Confidence interval
MD: Mediterranean Diet
MEDAS: Mediterranean Diet Adherence Screener
MEDLIFE: Mediterranean Lifestyle Index
ENDESA: National Demographic and Health survey
